# CD44-targeting hyaluronic acid-selenium nanoparticles boost functional recovery following spinal cord injury

**DOI:** 10.1186/s12951-024-02302-0

**Published:** 2024-01-23

**Authors:** Wenqi Luo, Yueying Li, Jianhui Zhao, Renrui Niu, Chunyu Xiang, Mingyu Zhang, Chunsheng Xiao, Wanguo Liu, Rui Gu

**Affiliations:** 1 Department of Orthopaedic Surgery, China-Japan Union Hospital of Jilin University, Changchun, 130033 People’s Republic of China; 2 Department of Hand and Foot Surgery, China-Japan Union Hospital of Jilin University, Changchun, 130033 People’s Republic of China; 3 Key Laboratory of Polymer Ecomaterials, Changchun Institute of Applied Chemistry, Chinese Academy of Sciences, Changchun, 130022 People’s Republic of China

**Keywords:** CD44 targeting, Inflammation, Reactive oxygen species, Selenium nanoparticles, Spinal cord injury

## Abstract

**Background:**

Therapeutic strategies based on scavenging reactive oxygen species (ROS) and suppressing inflammatory cascades are effective in improving functional recovery after spinal cord injury (SCI). However, the lack of targeting nanoparticles (NPs) with powerful antioxidant and anti-inflammatory properties hampers the clinical translation of these strategies. Here, CD44-targeting hyaluronic acid-selenium (HA-Se) NPs were designed and prepared for scavenging ROS and suppressing inflammatory responses in the injured spinal cord, enhancing functional recovery.

**Results:**

The HA-Se NPs were easily prepared through direct reduction of seleninic acid in the presence of HA. The obtained HA-Se NPs exhibited a remarkable capacity to eliminate free radicals and CD44 receptor-facilitated internalization by astrocytes. Moreover, the HA-Se NPs effectively mitigated the secretion of proinflammatory cytokines (such as IL-1β, TNF-α, and IL-6) by microglia cells (BV2) upon lipopolysaccharide-induced inflammation. In vivo experiments confirmed that HA-Se NPs could effectively accumulate within the lesion site through CD44 targeting. As a result, HA-Se NPs demonstrated superior protection of axons and neurons within the injury site, leading to enhanced functional recovery in a rat model of SCI.

**Conclusions:**

These results highlight the potential of CD44-targeting HA-Se NPs for SCI treatment.

**Supplementary Information:**

The online version contains supplementary material available at 10.1186/s12951-024-02302-0.

## Introduction

Spinal cord injury (SCI) is a catastrophic condition that results in severe disability, including paraplegia or quadriplegia, and gives rise to a range of serious multi-system complications, such as recurrent bladder and kidney infections, intestinal problems, as well as cardiac and respiratory dysfunction, placing a heavy burden on individuals, families, and society [1, 2]. In clinical practice, the conventional approaches for treating SCI encompass administration of high-dose methylprednisolone, implementation of surgical interventions aimed at stabilizing and decompressing the spinal cord, and provision of rehabilitative therapy [3, 4]. However, currently available therapies are mainly palliative and do not offer substantial functional recovery [5–8]. Hence, there is an impetus to explore alternative therapeutic strategies that consider the pathophysiology of SCI. The deleterious pathophysiological events that occur following SCI are the consequence of a biphasic process that encompasses both primary and secondary impairments [9–12]. The primary damage stems from the mechanical force and the initial impairment that subsequently initiates a rapidly escalating sequence of degenerative occurrences, commonly referred to as the secondary injury [13–15]. The secondary injury comprises various pathological events, such as the generation of reactive oxygen species (ROS) and the occurrence of inflammation [16–18]. In particular, overproduction of ROS at the injury site leads to the depletion of endogenous antioxidants and the disruption of redox balance, causing extensive damage to intracellular biomacromolecules, such as DNA and proteins, followed by consequent neuronal cell death [17, 19]. Moreover, excess ROS provoke an aggravated inflammatory response, recruiting and activating inflammatory cells, such as microglia and astrocytes, which further exacerbates neuronal cell death [14, 17]. Hence, it can be argued that ROS significantly contribute to the occurrence and progression of secondary injury in SCI.

It is well-documented that timely therapeutic intervention through ROS scavenging and inflammation suppression at the initial phase of SCI can significantly improve neurological and functional recuperation [14, 18, 20–23]. Recently, the utilization of nano-biomaterials as a therapeutic approach for SCI has garnered considerable interest [5, 15, 24–26]. Among them, selenium (Se)-based nanoparticles (NPs) have emerged as promising candidates due to their capacity for scavenging ROS and mitigating inflammation [27–29]. It has been proven that Se supplementation can forestall ROS accumulation, suppress lipid peroxidation, and prevent cellular damage [30–33]. Moreover, Se has been shown to mitigate functional deficits in the central nervous system due to its antioxidation properties [15, 34, 35]. Notwithstanding, conventional Se NPs have drawbacks, such as limited targeting ability, substandard stability, and limited solubility, which impede their application in basic research and the clinic [36]. It is therefore imperative to formulate innovative approaches to ensure the accumulation of Se NPs at the lesion site, favorable biological effects, and safety for therapeutic applications.

**Scheme 1:**
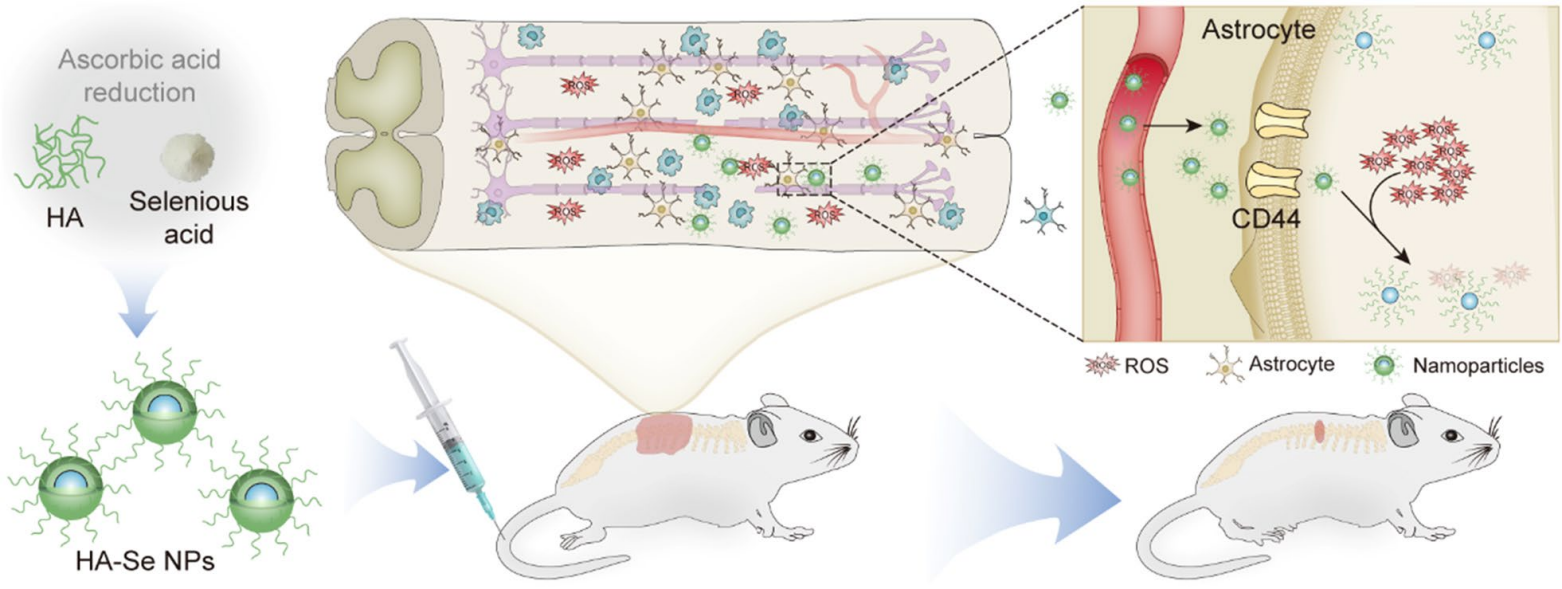
Schematic illustration of the preparation of CD44-targeting HA-Se NPs for promoting functional recovery after SCI

Hyaluronic acid (HA), a polysaccharide originating from the natural extracellular matrix, serves as a ligand for cluster of differentiation 44 (CD44) receptor [37]. Furthermore, it has been demonstrated that HA is beneficial for repairing spinal cord tissue by preventing the formation of glial scars [38]. In the current work, we focused the engineering and fabrication of HA-stabilized selenium nanoparticles (HA-Se NPs) specifically targeting the overexpressed CD44 receptor within the injured spinal cord, with the aim of mitigating secondary injury (Scheme 1). The HA-Se NPs were prepared via reduction of seleninic acid in the presence of sodium ascorbate. The obtained HA-Se NPs exhibited an exceptional ROS scavenging ability. Moreover, in vivo assessments confirmed that the HA-Se NPs tended to accumulate at the injury site through active binding to CD44, which is upregulated in activated astrocytes. By virtue of these targeting and ROS-scavenging abilities, the HA-Se NPs achieved superior neuroprotection and enhanced functional recovery in a rat model of SCI.

## Materials and methods

High-molecular-weight HA (with a molecular weight of 2 × 10^5^ g mol^−1^) and seleninic acid were procured from Huaxia Chemical Reagent Co., Ltd. (Chengdu, China). Sodium ascorbate was obtained from Aladdin Bio-Chem Technology Co., Ltd. (Shanghai, China). Dulbecco’s modified Eagle’s medium (DMEM) and fetal bovine serum were purchased from Gibco (Thermo Fisher Scientific, Waltham, MA, USA). 3-(4,5-dimethyl-thiazol-2-yl)-2,5-diphenyl tetrazolium bromide (MTT), 2′,7′-dichlorodihydrofluorescein diacetate (DCFH-DA; D6883), and 4′,6-diamidino-2-phenylindole dihydrochloride (DAPI) were purchased from Sigma-Aldrich (St. Louis, MO, USA). Hydrogen peroxide solution (30 wt% in water) was obtained from Sinopharm Chemical Reagent Co., Ltd. 1,1-Diphenyl-2-picrylhydrazyl (DPPH) was purchased from Shanghai Macklin Biochemical Co., Ltd. Anti-GFAP, anti-NF200, and anti-NeuN primary antibodies were obtained from Abcam (Cambridge, UK). Anti-CD44 and anti-caspase-3 antibodies were purchased from Cell Signaling Technology (Danvers, MA, USA). The dialysis membrane was obtained from Greenbird Technology Co., Ltd (Shanghai, China). The remaining compounds were obtained from commercial providers and used without any further manipulation.

### Preparation and characterization of HA-Se NPs

Briefly, 45 mL of aqueous HA solution (1.5 mg/mL) was mixed with 600 µL of seleninic acid (0.1 M) in a round-bottom flask. Subsequently, 3 mL of ascorbic acid aqueous solution (0.1 M) was gradually introduced into the mixture with constant stirring at ambient temperature (~ 22 °C). The gradual addition of sodium ascorbate solution resulted in a noticeable alteration of the solution’s color from a clear hue to a red tint. Upon completion of the reaction, the mixture was subjected to dialysis for 48 h using ultrapure water and a dialysis bag with a molecular weight cutoff (MWCO) of 300 kDa. Thereafter, the solution was centrifuged at 4,000 rpm for 20 min. Finally, the HA-Se NPs were obtained via lyophilization as red powder (yield: 82.5%).

The size and zeta potential of HA-Se nanoparticles were evaluated via dynamic light scattering on a Malvern Zetasizer Nano ZS (ZEN3600, Malvern Instruments, Worcestershire, UK). The morphology of HA-Se NPs was observed through transmission electron microscopy (TEM) (JOEL-1011, Tokyo, Japan) at an accelerating voltage of 200 kV and emission-scanning electron microscopy (SEM) (Zeiss, Oberkochen, Germany). Using the KBr pellet method, Fourier transform infrared spectra (FT IR) were recorded with a Win-IR device (Bio-Rad Laboratories, Hercules, CA, USA). The amount of selenium in HA-Se NPs was determined using an inductively coupled plasma mass spectrometer (ICP-MS, Xseries II, Thermo Scientific, USA). The antioxidant capacity of HA-Se NPs was explored by analyzing their influence on DPPH free radicals. In the control group, a combination of water (2 mL) and DPPH anhydrous ethanol solution (0.4 mM, 2 mL) was utilized. The experimental group, on the other hand, included HA-Se NPs (250/1, 000 µg/mL, 2 mL) and DPPH anhydrous ethanol solution (0.4 mM, 2 mL), while the blank group included HA-Se NPs (250/1,000 µg/mL, 2 mL) and absolute ethanol (2 mL). The background was modified through the utilization of a mixture of 2 mL of water and 2 mL of anhydrous ethanol. The aforementioned groups were exposed to darkness for a period of 30 min, after which the absorbance at 517 nm was recorded on a Bio-Rad 680 microplate reader (TECAN Trading AG, Switzerland). The free radical-scavenging rate (%) was determined as per the following formula: (1−(*A*_experimental_ − *A*_blank_)/*A*_control_) ×100, where *A*_*experimental*_, *A*_*blank*_, and *A*_*control*_ represent the absorbance values of the experimental, blank, and control group, respectively.

### Cytotoxicity and neuroprotective activity assay in vitro

Astrocytes, pheochromocytoma 12 (PC12) cells, and the BV2 microglia cell line were procured from the Cell Bank of the Chinese Academy of Science (Shanghai, China). The MTT method was employed to evaluate the cytotoxicity of HA-Se NPs in astrocytes and PC12 cells [22]. In brief, astrocytes or PC12 cells were seeded onto 96-well plates at a concentration of 8,000 cells per well and incubated for 12 h in DMEM medium. Subsequently, different concentrations of HA-Se NPs were added to the wells, ranging from 3.125 to 100 µg/mL. Control group cells were subjected to treatment with phosphate-buffered saline (PBS). Three parallel tests were carried out for every concentration. After a further incubation for either 24 or 48 h, solution containing 5 mg/mL MTT was added to each well at a volume of 20 µL and allowed to incubate for a further 4 h. Thereafter, the medium was discarded and exchanged with 150 µL of dimethyl sulfoxide. The absorbance of each well at 492 nm was determined using an absorbance microplate reader (Infinite M200, Tecan, Switzerland). In an effort to assess the potential protective properties of HA-Se NPs against H_2_O_2_-induced oxidative stress [22], astrocytes were grown in 96-well plates, with each well containing 8,000 cells. Prior to H_2_O_2_ exposure, the cells underwent 30-min pre-treatment with either PBS or HA-Se NPs at 50 and 100 µg/mL. Thereafter, the cells were exposed to 100 µM H_2_O_2_ for 24 h, and their viability was assessed via MTT assay. To further evaluate cell viability, we employed a commercial live-dead cell staining kit from Sigma-Aldrich. After staining, cells were observed through a confocal laser-scanning microscope (CLSM) (LSM 780, Zeiss). Dead cells were then quantified using the ImageJ software (1.51k, NIH, Bethesda, MD, USA). DCFH-DA fluorescence intensity was measured to quantitatively determine ROS levels in both astrocytes and PC12 cells.

### Assessment of proinflammatory cytokine levels in vitro and in vivo

In order to examine the capacity of HA-Se NPs to inhibit inflammation, BV2 cells were first seeded onto 6-well plates at a density of 2 × 10^5^ cells/well and permitted to settle for 12 h. Cells were then subjected to pre-treatment with either PBS or HA-Se NPs (20 µg/mL) for 2 h. Thereafter, the cells were exposed to 1 µg/mL lipopolysaccharide (LPS) for 24 h. To determine the levels of interleukin (IL)-1β, tumor necrosis factor α (TNF-α), and IL-6, we employed commercially available enzyme-linked immunosorbent assay kits (Anoric Bio-technology Co., Ltd) and an absorbance microplate reader (Infinite M200, Tecan, Switzerland). The assays were performed in triplicate. To confirm the in vivo suppressive effects of HA-Se NPs on inflammatory cells, which typically exhibit a peak at the lesion site within 3–7 days post-injury, immunofluorescence analysis of spinal cord tissue samples was carried out using anti-CD68 and anti-Iba-1 antibodies.

### Animal model of SCI

Approval for animal procedures was granted by the Animal Ethics Committee of Jilin University (approval No. SY202103013). Female Sprague-Dawley rats, aged 6–8 weeks, were procured from Liaoning Changsheng Biotechnology Ltd. and provided with *ad libitum* access to food and water. Sprague-Dawley rats were anesthetized via intraperitoneal administration of pentobarbital sodium intraperitoneally at a dose of 5 mg per kilogram of body weight. After disinfecting with iodine volts, a T10 laminectomy procedure was performed to reveal the spinal cord, and the injury model was established using a weight-drop device (C4P01-001, Shenzhen, China). A 40-g rod was allowed to fall from a height of 50 mm, making contact with the spinal cord’s uncovered dorsal surface, and causing an initial depth of penetration of 2.5 mm. Following the surgical intervention, the fascia, muscle, and skin were sequentially closed in layers. The Sprague-Dawley rats were housed in a temperature-regulated setting at 25 ± 2 °C and were provided with ad libitum access to both water and food. Moreover, the Sprague-Dawley rats were treated with cefazolin, given twice daily for a duration of 5 days at a dose of 25 mg/kg. The urinary bladders of Sprague-Dawley rats were manually emptied twice daily until they regained the ability to urinate autonomously.

The SCI rats were randomly divided into four groups (*n* = 9). The rats were treated with saline, HA-Se NPs (1 mg kg^−1^), HA-Se NPs (5 mg kg^−1^), or HA-Se NPs (10 mg kg^−1^). All rats received intravenous injections. The animals underwent anesthesia and were then perfused with 4% paraformaldehyde and PBS at the 12-week interval post-injury. After being harvested from the center of injury, the spinal cords, which had a length of roughly 2 cm, were treated with a 4% paraformaldehyde solution. Thereafter, the specimens were embedded in paraffin for further analysis. These sections were employed in subsequent experiments.

### Analysis of CD44 expression

CD44 expression in the spinal cord was evaluated seven days after injury. The tissue slices underwent dual labeling using a primary anti-CD44 antibody in conjunction with primary anti-glial fibrillary acidic protein (GFAP), anti-NeuN, anti-CD68, or anti-Iba-1 antibodies. For immunofluorescence staining, the antibody utilized is documented in Additional file 1: Table S1 . CLSM was utilized to obtain images. Immunofluorescence experiments were performed three times. To examine the potential for inflammation to stimulate CD44 expression in astrocytes, the cells were grown on 6-well plates and subjected to varying concentrations of LPS (10, 20, or 40 µg/mL) or glutamate (100, 200, or 400 µM) for a duration of 24 h. CD44 expression was evaluated via western blot and immunofluorescence analyses. Following LPS activation for 24 h, astrocytes were rinsed with PBS and were then fixed with 4% PFA. Nonspecific binding sites were obstructed using blocking buffer for a period of 1.5 h. Thereafter, the cells were incubated with an anti-CD44 primary antibody and incubated overnight at a temperature of 4 °C. After being washed with PBS, the cells were exposed to secondary antibodies and incubated for a duration of 2 h at room temperature. Another PBS wash was then carried out, and the cells were subsequently exposed to 1,1′-dioctadecyl-3,3,3′,3′-tetramethylindocarbocyanine perchlorate (Dil), a fluorescent probe for cell membranes, for a duration of 15 min. Ultimately, the astrocytes were examined and visualized via CLSM. This experiment was also repeated three times.

To ascertain the potential association between the CD44 receptor and the absorption of HA-Se NPs, a competitive cellular uptake experiment was performed. Following a 24-h incubation with LPS, astrocytes were subjected to a 4-h pretreatment with either HA (1 mg/mL) or PBS. Samples were then subjected to incubation with 0.1 mg/mL HA-Se NPs for 4 and 8 h, followed by five washes with PBS and fixation with 4% PFA for a period of 20 min. Cell nuclei were stained with DAPI staining prior to observation via confocal microscopy (LSM 780, Zeiss, Germany). Mean intracellular fluorescence intensity was determined using ImageJ (1.51k, NIH, Bethesda, MD, USA).

### Biodistribution of HA-Se NPs

NH_2_-Cy5-labeled HA-Se NPs were synthesized as previously described [39]. To visualize the in vivo biodistribution of HA-Se NPs, rats were administered Cy5-labeled HA-Se NPs at a dose of 10 mg/kg subsequent to the injury. At specific intervals (4, 12, and 24 h), the rats were humanely sacrificed, and their primary organs (heart, lung, liver, spleen, brain, kidney, and spinal cord) were obtained. Control samples were obtained by administering Cy5-labeled HA-Se NPs to sham rats, which were then euthanized 12 h post-injection. The obtained organs were imaged and analyzed using a Maestro In Vivo Imaging System (IVIS Lumina LT Series III, PerkinElmer, USA).

### Behavioral analysis of SCI rats

The Basso, Beattie, Bresnahan (BBB) locomotor rating scale was employed to evaluate the motor functions of rats. Based on the 21-point BBB open-field grading scale, rats were individually assessed in an open field for 5 min. The BBB score assesses the voluntary movements of rat limbs through a scale of 0 to 21. A score of 0 represents paralysis, whereas 21 indicates normal movements. Evaluation was performed by two separate evaluators, and the marks were ascertained through mutual accord.

### Histological and immunofluorescence analyses

Fixed spinal cord samples were embedded in paraffin, followed by sectioning into 4 μm thick slices in the coronal plane. Through H&E staining, pathological changes, which included enlargement of cavity area and inflammatory cell infiltration, were investigated. The extent of demyelination was determined by staining spinal cord slices from each group with 0.1% Luxol fast blue (LFB) at the 12-week mark following injury.

To evaluate the myelin sheath ultrastructure, we prepared tissue for transmission electron microscopy (TEM) as per our previously established method [22]. The spinal cord segments were fixed overnight at a temperature of 4 °C using 2.5% glutaraldehyde. Thereafter, the segments were sliced into 1 mm^3^ pieces, osmicated for 90 min, and then dehydrated for a period of 135 min. Subsequently, the ultrathin sections were subjected to staining with uranyl acetate and lead citrate, followed by a thorough examination using TEM. To assess the expression of neurofilament 200 (NF200) and NeuN, the slices were first subjected to permeation with a PBS solution that contained 0.1% Triton X-100 for a period of 15 min. Thereafter, a 2-h incubation period with 5% bovine serum albumin (BSA) was utilized for blocking, followed by thorough washing with PBS containing 0.2% Tween 20. Subsequently, the portions were subjected to an overnight incubation at a temperature of 4 °C, utilizing primary antibodies against NF200 and NeuN. The axons and neurons were distinguished using anti-NF200 and anti-NeuN, respectively. Additional file 1: Table S1 provides details on antibody dilution for immunofluorescence. Images were acquired through CLSM. The potential toxicity of HA-Se NPs was assessed 12 weeks post-treatment through H&E staining of vital organs.

### Western blotting

Spinal cord tissue was lysed using a buffer solution that contained proteinase inhibitors. The lysate was then centrifuged at 13,300 rpm for 15 min. Protein concentration was determined using a BCA protein kit (Beyotime, Shanghai, China). The procedure involved separation of 10 µg protein from each sample using sodium dodecyl sulfate-polyacrylamide gel electrophoresis (SDS-PAGE) and subsequent transfer of the separated protein onto polyvinylidenedifluoride (PVDF) membranes (EMD Millipore, Billerica, MA, USA). For blocking, the membranes were treated with 5% bovine serum albumin for 1.5 h. Thereafter, membranes were incubated with either anti-CD44 or anti-caspase-3 antibodies at 4 °C overnight. After washing, the membranes were incubated with a secondary antibody conjugated to horseradish peroxidase for 2 h at room temperature. Target protein expression was normalized based on that of GAPDH or β-actin. Western blotting antibody data is available in Additional file 1: Table S2. An enhanced chemiluminescence Western blot detection system (AI600 Imager; GE Healthcare, Chicago, IL, USA) was used to capture protein bands. Densitometric analysis of the protein bands was carried out using Multi Gauge software (Fuji, Tokyo, Japan). Western blotting experiments were repeated three times.

### Statistical analysis

To determine the statistical significance of differences, a one-way analysis of variance or t-tests were performed utilizing Prism 8.0.2 (GraphPad Software, San Diego, CA, USA). *P < 0.05 was deemed statistically significant. **P < 0.01 and ***P < 0.001 were deemed highly significant.

## Results

### Preparation and characterization of HA-Se NPs

**Fig. 1:**
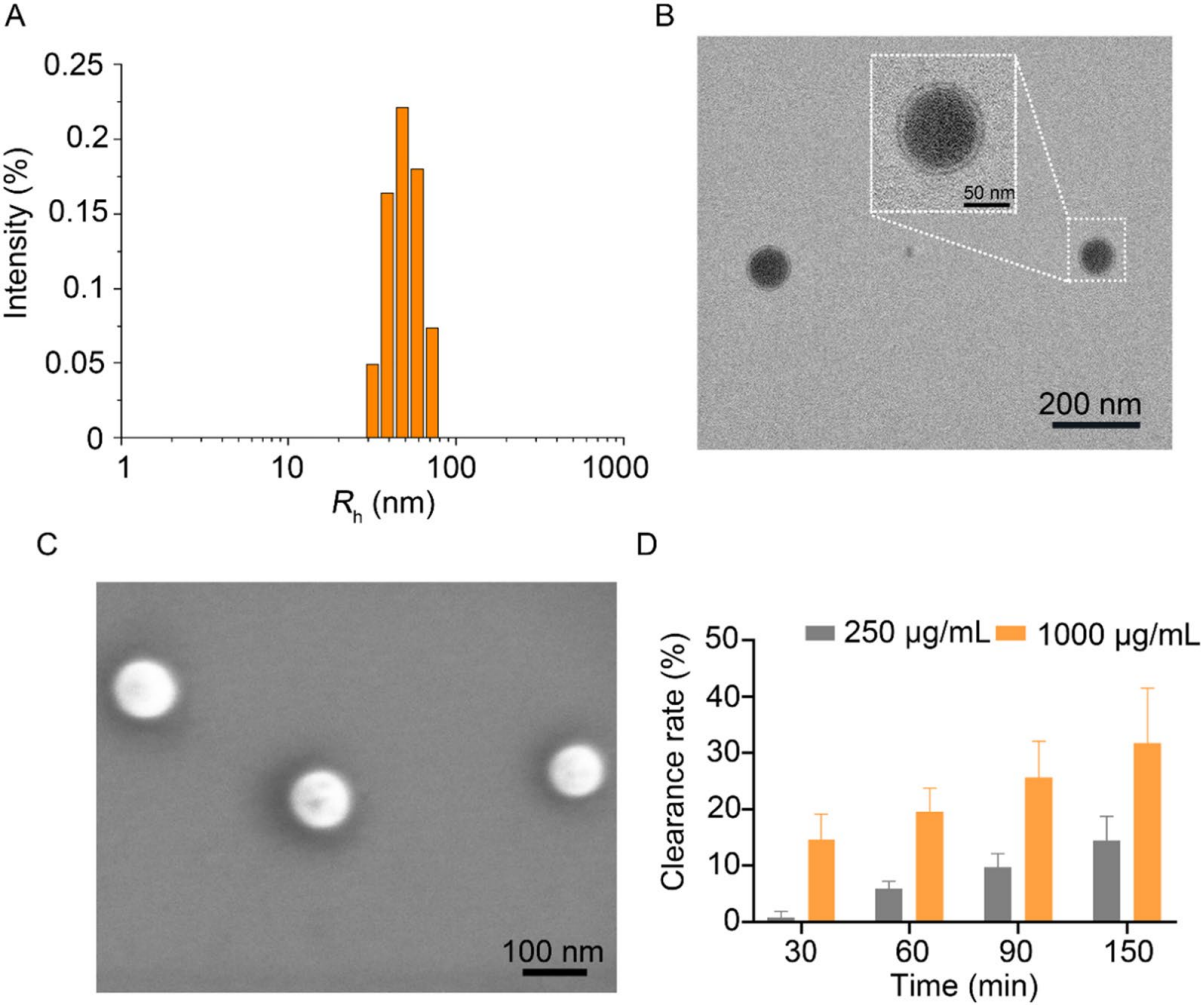
Characterization of hyaluronic acid-selenium nanoparticles (HA-Se NPs). **A** Dynamic light scattering measurement of HA-Se NPs. **B** Transmission and **C** scanning electron microscopy imaging of HA-Se NPs. **D** The efficacy of HA-Se NPs in scavenging free radicals was evaluated at varying concentrations

The synthesis of HA-Se NPs was accomplished through a straightforward reduction reaction, utilizing water-soluble HA as a stabilizing and capping agent [40]. The HA acts as a capping agent to facilitate NP formation and also provides a protective shell to prevent NP aggregation. The HA-Se NPs exhibited a size of 101.2 ± 1.4 nm and a polydispersity index of 0.17 ± 0.02 (Fig. 1A). The particle size of HA-Se NPs did not change significantly over a period of 3 days (Additional file 1: Figure S1), which indicates a high degree of stability. Through TEM, we observed that HA-Se NPs possessed a core-shell structure, with a diameter averaging roughly 95 nm (Fig. 1B and Additional file 1: Figure S2). Moreover, SEM analysis revealed that HA-Se NPs exhibited a uniform spherical morphology, with an approximate size of 94 nm (Fig. 1C). The zeta potential of HA-Se NPs was − 26.2 ± 0.47. Their composition was investigated in greater detail using X-ray photoelectron spectroscopy, which confirmed that the nanoparticles were predominantly composed of carbon, nitrogen, oxygen, and selenium, as indicated by the distinctive peaks detected in the C 1s, N 1s, O 1s, and Se 3d spectra. (Additional file1: Figure S3). The selenium content of HA-Se NPs was 10.8%, as determined via ICP-MS. As oxidative stress can cause significant and continuous harm to the central area and adjacent spinal cord [6, 24], the capacity of HA-Se NPs to eliminate ROS was subsequently examined. HA-Se NPs exhibited remarkable ability in eliminating DPPH free radicals that was concentration- and exposure duration-dependent, as shown in Fig. 1D.

### Biocompatibility of HA-Se NPs, protection from H_2_O_2_-induced oxidative stress, and inflammation suppression in vitro

**Fig. 2:**
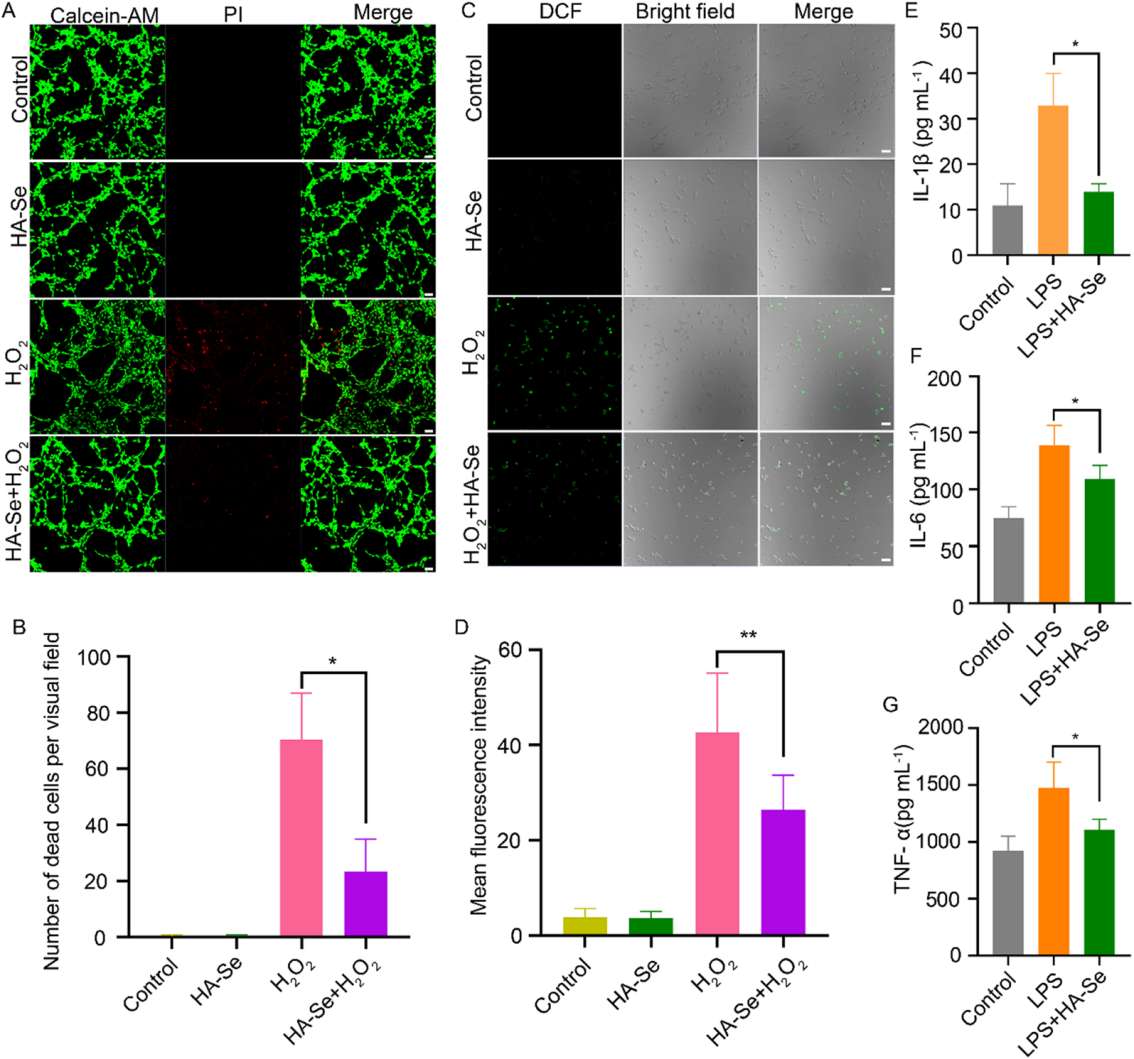
The efficacy of HA-Se NPs in preventing H_2_O_2_-induced oxidative stress and inflammation was evaluated. **A** Live/dead staining of astrocytes. Scale bar = 20 μm. The concentration of H_2_O_2_ was 100 µM. **B** Quantitative analysis of astrocyte cell death. *P < 0.05. **C** Levels of reactive oxygen species within astrocytes were determined via DCFH-DA staining. **D** Quantitative analysis of DCF fluorescence intensity in astrocytes. **P < 0.01. Quantification of **E** interleukin (IL)-1β, **F** IL-6, and **G** tumor necrosis factor (TNF)-α levels in BV2 cell culture medium in the presence or absence of HA-Se NPs. *P < 0.05

The biocompatibility of the synthesized HA-Se NPs was investigated through MTT tests. The impact of HA-Se NPs on astrocytes and PC12 cells was evaluated through observation and analysis after 24 or 48 h of exposure at concentrations ranging from 0 to 100 mg/L. Notwithstanding the highest concentration tested, the HA-Se NPs induced no apparent cytotoxicity, as shown in Additional file 1: Figure S4. Oxidative stress occurs when excessive ROS are generated after SCI [41–43]. HA-Se NPs successfully protected astrocytes from oxidative stress induced by H_2_O_2_, preventing cell death (Figure S5). Live/dead cell staining further confirmed the protective effect of HA-Se NPs (Fig. 2A, B and Additional file 1: Figure S6A, B). Consistently, the presence of HA-Se NPs effectively reduced the amount of ROS during in vitro culture of astrocytes and PC12 cells, as indicated by the reduced fluorescence intensity of a ROS probe (Fig. 2C, D and Additional file 1: Figure S6C, D). The anti-inflammatory efficacy of HA-Se NPs was determined in BV2 cells exposed to LPS (1 µg/mL). Incubation with HA-Se NPs completely reversed the LPS-induced increase in primary cytokines (TNF-α, IL-1β, and IL-6) (Fig. 2E–G).

### Targeting efficiency of HA-Se NPs in vitro and in vivo

**Fig. 3:**
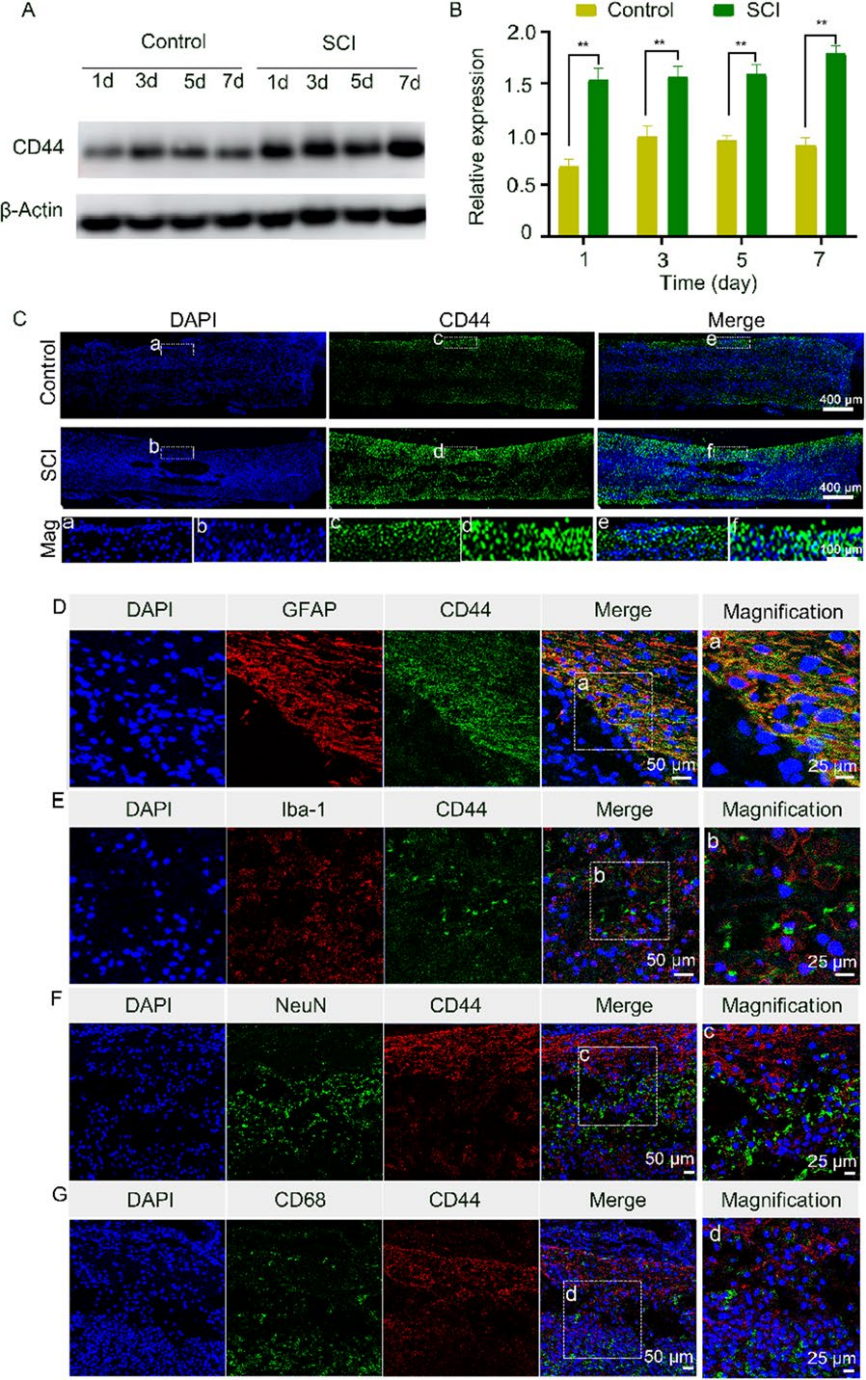
CD44 expression in the injured spinal cord. **A** Representative western blot images of CD44 expression in the control and SCI groups. **B** Densitometric analysis of CD44 levels based on data in (**A**). **P < 0.01 in comparison to the control group. **C** CD44 immunofluorescence staining of injured spinal cord samples. Mag: Magnification. **D** Immunofluorescence staining of injured spinal cord samples for GFAP (red) and CD44 (green). **E** Immunofluorescence staining of injured spinal cord samples for CD44 (green) and Iba-1 (red). **F** Immunofluorescence staining of injured spinal cord samples for NeuN (green) and CD44 (red). **G** Immunofluorescence staining of injured spinal cord samples for CD68 (green) and CD44 (red)

Insufficient information exists regarding CD44 expression within the injured spinal cord [44]. Thus, we evaluated CD44 expression in the injured spinal cord of rats. Increases in CD44 levels were detected at 1-, 3-, 5-, and 7-days post-injury (Fig. 3A–C). To further characterize the cells which exhibited CD44 upregulation, we performed immunofluorescence staining. As indicated in Fig. 3D and E, GFAP and CD44 signals completely co-localized, while Iba-1 and CD44 exhibited partial co-localization. In addition, CD68 and CD44 fluorescence signals as well as those of NueN and CD44 exhibited incomplete colocalization (Fig. 3F and G). Taken together, these findings suggest that CD44 was expressed in astrocytes and a subset of microglial cells.

**Fig. 4:**
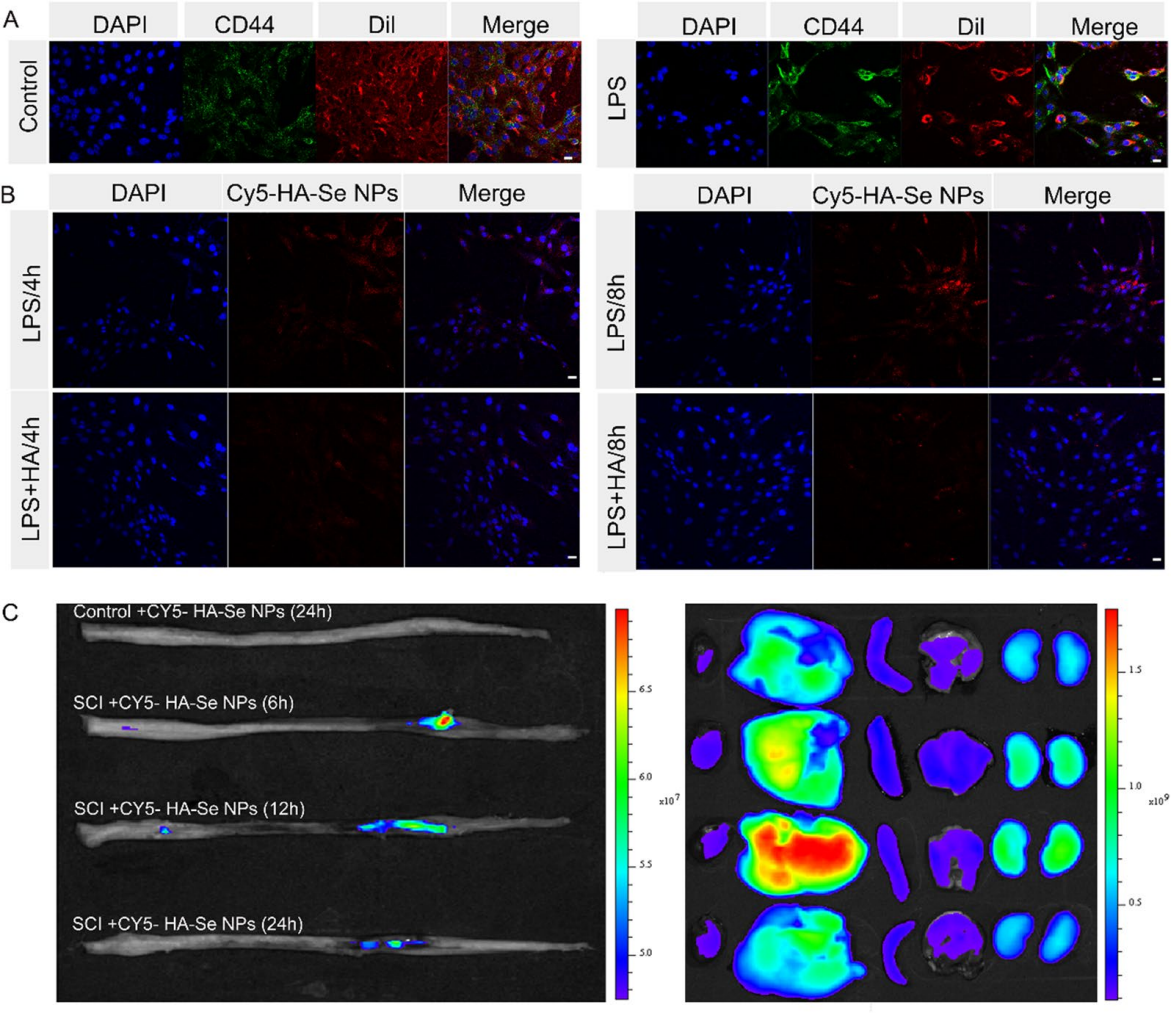
CD44 immunofluorescence staining in astrocytes and internalization of Cy5-HA-Se NPs. **A** Astrocytes stained for CD44 and Dil. Scale bar = 50 μm. **B** Cy5-HA-Se NPs internalization by astrocytes after 4 and 8 h of incubation. Scale bar = 50 μm. **C** Targeting efficiency of HA-Se NPs in vivo. Fluorescence images of the major organs after intravenous injection of rats with Cy5-labeled HA-Se NPs. The organs were collected at 24 h post-injection from non-treated rats and at 6, 12, and 24 h post-injection from SCI rats

It has been previously established that an inflammatory microenvironment, such as that induced by LPS, promotes the secretion of proinflammatory cytokines by microglial cells and astrocytes [45–48]. Further, research has shown that is CD44 upregulated during the inflammatory response [49–51]. Thus, we exposed astrocytes to LPS for 24 h, observing notably enhanced CD44 expression in the LPS group as compared to controls, indicative of a substantial elevation in CD44 following LPS activation (Fig. 4A and Additional file 1: Figure S7). Western blotting confirmed the upregulation of CD44 in LPS-stimulated astrocytes (Additional file 1: Figure S8A, B). Conversely, the presence of glutamate did not alter CD44 expression in astrocytes, as indicated by the data presented in Additional file 1: Figure S8C and D.

Next, we explored the targeting efficiency of HA-Se NPs in vitro, using LPS to induce CD44 expression in astrocytes. The time-dependent internalization of HA-Se NPs by astrocytes was confirmed based on a gradual increase in red fluorescence, which we attributed to CD44 expression. To corroborate this observation, we blocked CD44 with HA [37, 52, 53]. In LPS-activated astrocytes, time-dependent internalization was observed in both the HA pretreatment and non-pretreatment groups, being stronger in the latter group during the initial 4 and 8 h (Fig. 4B and Additional file 1: Figure S9). These results validated that enhanced HA-Se NPs internalization was achieved via CD44. To assess NP targeting efficacy in vivo, rats were administered Cy5-labeled HA-Se NPs after SCI. Following injection, the injured spinal cord exhibited a high fluorescent signal within a mere 6-h window, with the fluorescent signal remaining strong at 12 h and subsequently waning at 24 h, possibly due to deterioration of the HA-Se NP shell structure. Cy5-labeled HA-Se NPs accumulated in the heart, liver, spleen, lungs, and kidneys, particularly in the liver and kidneys, which may suggest that the NPs are metabolized via these two organs (Fig. 4C). The fluorescence signal of the liver was gradually increased and reached to maximum at 12 h post-injection.

### Neuroprotective activity of HA-Se NPs in vivo

**Fig. 5:**
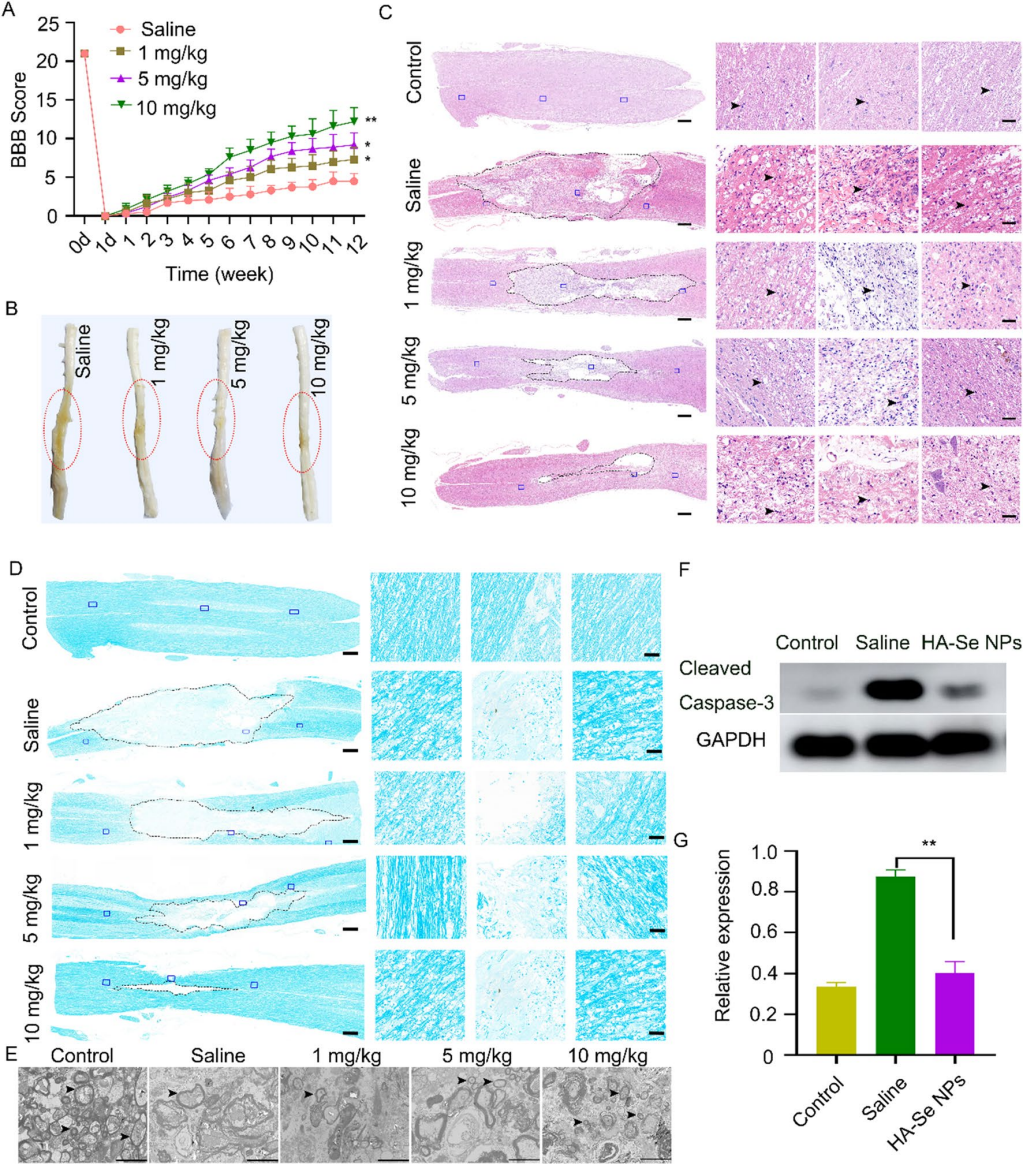
Neuroprotective effects of HA-Se NPs. **A** Basso Beattie Bresnahan (BBB) scores of SCI rats. **B** Gross images of the spinal cord in different groups at 12 weeks post-injury. The lesion site is denoted by the red circle. **C** Images of hematoxylin and eosin (H&E) right, three magnified images of the areas marked by the blue squares. Scale bars = 500 and 50 μm, respectively. Dotted line and arrows indicate the lesion cavity and inflammatory cells, respectively. **D** Images of an injured spinal cord stained with LFB. Scale bars = 500 and 50 μm, respectively. Dotted line indicates the lesion cavity. **E** Representative TEM images of different experimental groups after treatment with saline or HA-Se NPs at the indicated concentrations. Scale bar = 2 μm. Arrows indicate myelin sheaths. **F** Western blot analysis of cleaved caspase-3 in injured spinal cord tissues 24 h after injury. GAPDH was used as the internal control. **G** Densitometric analysis of cleaved caspase-3 levels based on (D). **P < 0.01 in relation to the saline group

To investigate the neuroprotective activity of HA-Se NPs in vivo, BBB scores were utilized to evaluate the restoration of locomotor function over a 12-week post-injury duration. The recovery of locomotor function was notably accelerated in all treatment groups as opposed to the saline group within a 12-week period following SCI. The group receiving 10 mg kg^−1^ exhibited a notably elevated BBB score compared to the other groups (Fig. 5A). This observation is also supported by visual inspection of SCI rat spinal cord tissues (Fig. 5B). Assessment of the pathological alterations that transpired at the site of the lesion revealed that the condition of spinal cords was notably inferior in the saline group, with greater numbers of inflammatory cells detected. In contrast, the animals receiving HA-Se NPs exhibited varying degrees of improvement in spinal cord integrity. Figure 5C illustrates that the animals receiving 10 mg/kg HA-Se NPs displayed the least extensive lesion cavity, with a sparse presence of inflammatory cells (Additional file 1: Figure S10). Based on the aforementioned observations, it can be inferred that the application of HA-Se NPs was effective in halting the progression of secondary injury cascades.

Axonal demyelination is a common result of SCI [6, 54, 55]. In light of this, we utilized LFB staining to assess the degree of myelination in axons. As indicated by Fig. 5D, there was a reduction in the number of intact myelin sheaths in the group treated with saline. HA-Se NPs treatment alleviated the severity of demyelination, indicating that HA-Se NPs preserved myelin sheaths following injury. To further verify changes in myelination, we performed TEM analysis of the myelin sheaths. In the saline-treated group, TEM images indicated that the myelin sheaths were disorganized and not compactly arranged (Fig. 5E). Meanwhile, the group treated with HA-Se NPs demonstrated greater amount, size, and thickness of myelin sheaths, with the best outcomes observed in rats treated with 10 mg/kg of HA-Se NPs.

**Fig. 6:**
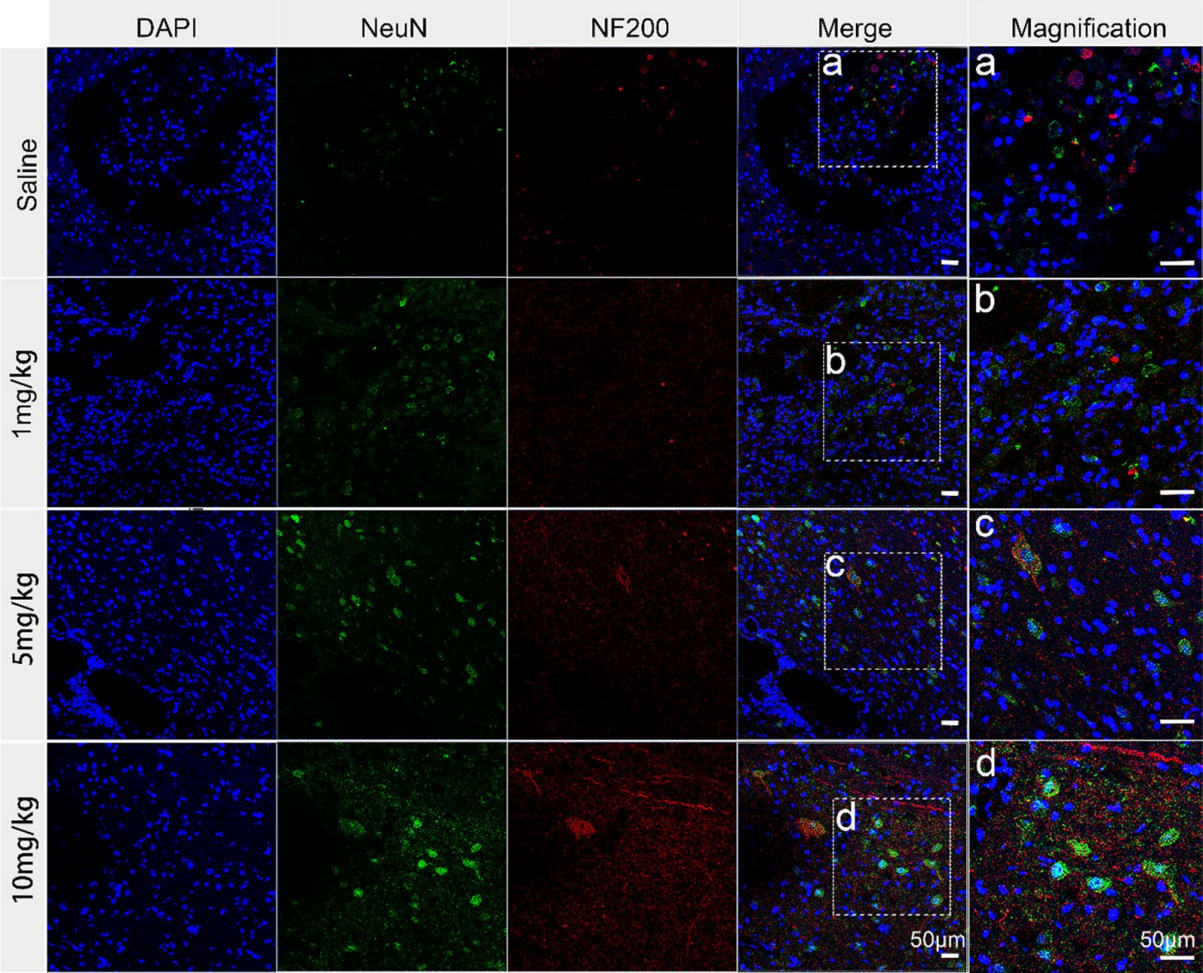
Immunohistochemistry of scar tissue labeled with NeuN (green) and NF200 (red). The group that received a dosage of 10 mg/kg exhibited greater preservation of both neurons and axons within the affected spinal cord tissue, even at 12 weeks post-SCI. Scale bar = 50 μm

It is worth mentioning that the application of HA-Se NPs brought about a significant reduction in the levels of cleaved caspase-3, which were significantly elevated following SCI (Fig. 5F, G). Western blot confirmed the significant reduction in cleaved caspase-3 levels within the spinal cords of animals treated with HA-Se NPs in comparison to those treated with saline. Subsequently, we assessed the remedial impacts of HA-Se NPs on both neurons and axons. Saline-treated controls exhibited a substantial loss of axons and neurons, as observed via confocal imaging. Conversely, animals administered different concentrations of HA-Se NPs displayed greater myelin sheath and axon retention, which highlighted the protective potential of HA-Se NPs in the affected area (Fig. 6). This outcome is consistent with the observed improvement in locomotive function (Fig. 5A). In addition, a considerable number of CD68- and Iba-1-positive cells (macrophage/microglia) were observed at the injury site in the saline group. In comparison, the HA-Se NPs group exhibited a notable decrease in CD68- and Iba-1-positive cells (Additional file 1: Figure S11). Taken together, these observations supported the therapeutic value of HA-Se NPs in enhancing locomotor function recovery through the preservation of neurons and axons. It should also be noted that the major organs obtained from rats administered HA-Se NPs exhibited no visible alterations (Additional file 1: Figure S12). It can therefore be inferred that HA-Se NPs are safe and effective for the management of SCI.

## Discussion

In the present study, we employed a facile redox approach to prepare the HA-Se NPs that specifically target CD44 receptor. HA-Se NPs scavenged ROS and restricted inflammatory responses. Further, HA-Se NPs efficiently accumulated at lesions by targeting CD44. Consequently, HA-Se NPs protected axons and neurons within the injury site, improving functional recuperation in a rat model of SCI. Therefore, we believe that CD44-targeting HA-Se NPs represent a viable therapeutic option for the treatment of SCI.

Therapeutic approaches that involve scavenging ROS and inhibiting inflammatory cascades within the damaged spinal cord have demonstrated efficacy in enhancing functional recovery following SCI [56, 57]. In the preliminary in vitro experiments, HA-Se NPs effectively scavenged DPPH free radicals, in a manner dependent on concentration and exposure time. To explore the capacity of HA-Se NPs for reducing ROS at the cellular level, H_2_O_2_ was used to mimic oxidative stress. HA-Se NPs significantly reduced ROS levels during in vitro culture of astrocytes and PC12 cells, in addition to reducing cell death. The damaged and surrounding spinal cord tissues are subject to infiltration by microglia/macrophages, which subsequently secrete proinflammatory cytokines, shaping a hostile microenvironment that hinders SCI repair [58–60]. Our results showed that HA-Se NPs dramatically suppress LPS-induced inflammatory cytokine (TNF-α, IL-1β, and IL-6) production. The anti-inflammatory capacity of HA-Se NPs was further demonstrated in our rat SCI model, based on a reduction in CD68- and Iba-1-positive cell infiltration. Taken together, these observations highlight the protective effect of HA-Se NPs, which shield spinal cord cells from oxidative stress-induced injury and aberrant inflammation.

Excessive ROS generation and the consequent aggressive inflammatory response are known to impede SCI repair [18]. Therefore, a prompt therapeutic intervention that involves the elimination of ROS and suppression of inflammation can yield substantial benefits in terms of sustained neurological and functional recovery [14]. In addition, targeting ability is an essential aspect in the enhanced curative effect and lesser side effects associated with NPs [61]. Our results showed that the CD44 receptor was highly expressed in astrocytes and microglia following SCI, in accordance with previous research [44, 62]. HA is a well-known ligand of the CD44 receptor [63]. Through series in vitro studies, we found that the HA-Se NPs were effectively internalized by astrocytes. In addition, the HA-Se NPs effectively accumulated within damaged spinal cord tissue, where they persist for a duration of 24 h. The positive impact of HA-Se NPs may be attributed to their exceptional targeting capability, which enables prolonged accumulation within spinal cord tissue.

## Conclusions

Herein, we developed CD44-targeting HA-Se NPs for enhanced SCI recovery through ROS elimination and the suppression of inflammatory responses. Upon SCI, astrocytes and certain microglial cells exhibit upregulated CD44 expression, which facilitate the accumulation HA-Se NPs and consequent protection of axons and neurons within the injury site. The aforementioned observations underscore the auspicious therapeutic potential of HA-Se NPs in oxidative stress-induced disease or their viability as potential nanocarriers for the treatment of SCI.

### Supplementary Information

**Additional file 1: Figure S1.** The stability of HA-Se NPs in pH 7.4 PBS buffer. **Figure S2.** Transmission electron microscopy (TEM) image of HA-Se NPs revealing a spherical shape, with a mean diameter of approximately 95 nm. **Figure S3.** X-ray photoelectron spectrum of hyaluronic acid-selenium nanoparticles (HA-Se NPs). **Figure S4.** *In vitro* biocompatibility of HA-Se NPs. Viability of PC12 cells and astrocytes incubated with different concentrations of HA-Se NPs for (A) 24 and (B) 48 h. Data are presented as mean ± SD (*n *= 3 for each group). **Figure S5.** The HA-Se nanoparticles scavenge reactive oxygen species (ROS) to protect astrocytes from oxidative damage. [H_2_O_2_] =100 μM. **Figure S6.** HA-Se NPs scavenge ROS to protect PC12 cells *in vitro*. (A) Live/dead staining of PC12 cells. Scale bar = 20 μm. (B) Quantitative analysis of dead cells. (C) Intracellular ROS levels in PC12 cells were measured using DCFH-DA staining. Scale bar = 20 μm. (D) Quantitative analysis of DCF fluorescence intensity in the cells. **P<0.01. **Figure S7.** Quantitative analysis of the mean fluorescence intensity of CD44 staining in Fig. 4A. **P<0.01 compared to the control group. **Figure S8.** Astrocytes overexpress CD44 upon LPS exposure. Western blot analysis of CD44 in astrocytes upon (A) LPS and (C) glutamate activation. (B, D) Densitometric analysis of CD44 levels based on the data in (A) and (C), respectively. *P<0.05, **P<0.01. **Figure S9.** Quantitative analysis of the mean fluorescence intensity of Cy5-HA-Se NPs in Fig. 4B. **P<0.01 in comparison to the LPS/8 h group. **Figure S10.** Quantitative analysis of inflammatory cells in Figure 5C,* P < 0.05, in the saline group compared with the 10 mg/kg group. **Figure S11.** Immunohistochemistry staining of scar tissue labeled with Iba-1 (green) and CD68 (red) 12 weeks after SCI. Scale bar = 50 μm. **Figure S12.** Hematoxylin & eosin (H&E) staining of the major organs in the experimental group. **Table S1.** Information on the antibodies used for immunofluorescence (IF) staining. **Table S2.** Antibodies used for western blotting (WB).

## Data Availability

No datasets were generated or analysed during the current study.

## Abbreviations

BBB: Basso Beattie Bresnahan
BSA: Bovine serum albumin
CLSM: Confocal laser-scanning microscope
DMEM: Dulbecco’s modified Eagle’s medium
LPS: Lipopolysaccharide
GFAP: Glial fibrillary acidic protein
H&E: Hematoxylin and eosin
HA: Hyaluronic acid
PBS: Phosphate-buffered saline
ROS: Reactive oxygen species
SCI: Spinal cord injury
SEM: Scanning electron microscopy
TEM: Transmission electron microscopy
NPs: Nanoparticles
