# Supplementary material for: CD44-targeting hyaluronic acid-selenium nanoparticles boost functional recovery following spinal cord injury
Source: J Nanobiotechnology. 2024 Jan 23;22:37. doi: 10.1186/s12951-024-02302-0 (PMC10804833; doi:10.1186/s12951-024-02302-0)
Supplement: Supplementary file 1 — Additional file 1: Figure S1. The stability of HA-Se NPs in pH 7.4 PBS buffer. Figure S2. Transmission electron microscopy (TEM) image of HA-Se NPs revealing a spherical shape, with a mean diameter of approximately 95 nm. Figure S3. X-ray photoelectron spectrum of hyaluronic acid-selenium nanoparticles (HA-Se NPs). Figure S4. In vitro biocompatibility of HA-Se NPs. Viability of PC12 cells and astrocytes incubated with different concentrations of HA-Se NPs for (A) 24 and (B) 48 h. Data are presented as mean ± SD (n = 3 for each group). Figure S5. The HA-Se nanoparticles scavenge reactive oxygen species (ROS) to protect astrocytes from oxidative damage. [H2O2] =100 μM. Figure S6. HA-Se NPs scavenge ROS to protect PC12 cells in vitro. (A) Live/dead staining of PC12 cells. Scale bar = 20 μm. (B) Quantitative analysis of dead cells. (C) Intracellular ROS levels in PC12 cells were measured using DCFH-DA staining. Scale bar = 20 μm. (D) Quantitative analysis of DCF fluorescence intensity in the cells. **P<0.01. Figure S7. Quantitative analysis of the mean fluorescence intensity of CD44 staining in Fig. 4A. **P<0.01 compared to the control group. Figure S8. Astrocytes overexpress CD44 upon LPS exposure. Western blot analysis of CD44 in astrocytes upon (A) LPS and (C) glutamate activation. (B, D) Densitometric analysis of CD44 levels based on the data in (A) and (C), respectively. *P<0.05, **P<0.01. Figure S9. Quantitative analysis of the mean fluorescence intensity of Cy5-HA-Se NPs in Fig. 4B. **P<0.01 in comparison to the LPS/8 h group. Figure S10. Quantitative analysis of inflammatory cells in Figure 5C,* P < 0.05, in the saline group compared with the 10 mg/kg group. Figure S11. Immunohistochemistry staining of scar tissue labeled with Iba-1 (green) and CD68 (red) 12 weeks after SCI. Scale bar = 50 μm. Figure S12. Hematoxylin & eosin (H&E) staining of the major organs in the experimental group. Table S1. Information on the antibodies used for immunofluorescence (IF) stai [file 12951_2024_2302_MOESM1_ESM.docx]

**Additional Information**

CD44-targeting hyaluronic acid-selenium nanoparticles boost functional recovery following spinal cord injury

Wenqi Luo^a#^, Yueying Li^b#^, Jianhui Zhao^a^, Renrui Niu^a^, Chunyu Xiang^a^, Mingyu Zhang^a^, Chunsheng Xiao^c^, Wanguo Liu^a^*, and Rui Gu^a^*

1. Department of Orthopaedic Surgery, China-Japan Union Hospital of Jilin University, Changchun, 130033, People’s Republic of China
2. Department of Hand and Foot Surgery, China-Japan Union Hospital of Jilin University, Changchun 130033, P. R. China
3. Key Laboratory of Polymer Ecomaterials, Changchun Institute of Applied Chemistry, Chinese Academy of Sciences, Changchun 130022, People’s Republic of China

# Wenqi Luo and Yueying Li contributed equally to this work.

* Correspondences: Rui Gu, gurui@jlu.edu.cn; Wanguo Liu, liuwanguo6016@jlu.edu.cn


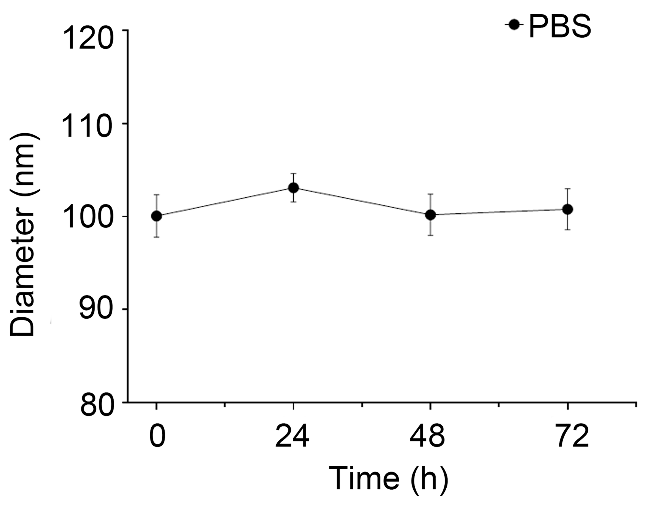


Figure S1. The stability of HA-Se NPs in pH 7.4 PBS buffer.


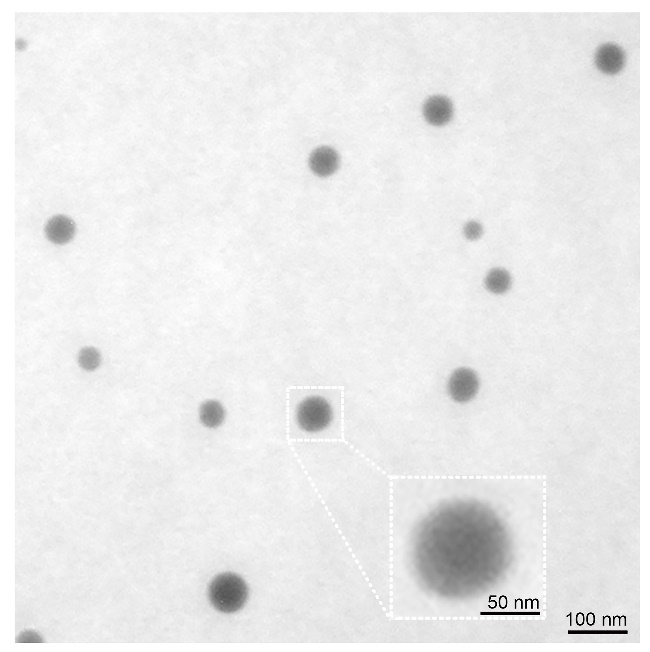


Figure S2. Transmission electron microscopy (TEM) image of HA-Se NPs revealing a spherical shape, with a mean diameter of approximately 95 nm.


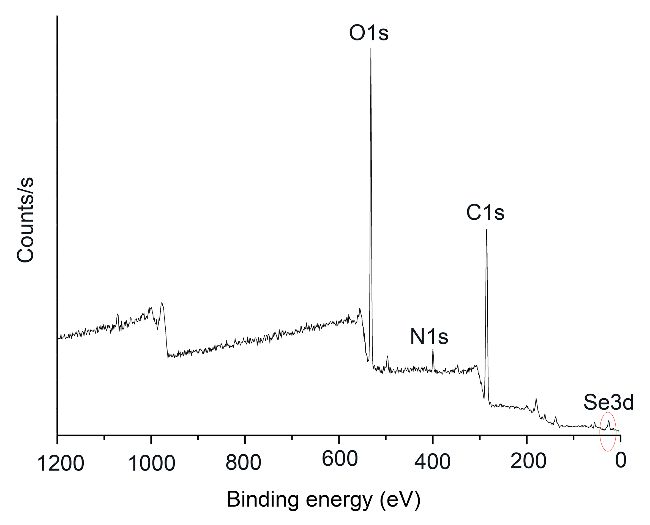


Figure S3. X-ray photoelectron spectrum of hyaluronic acid-selenium nanoparticles (HA-Se NPs).


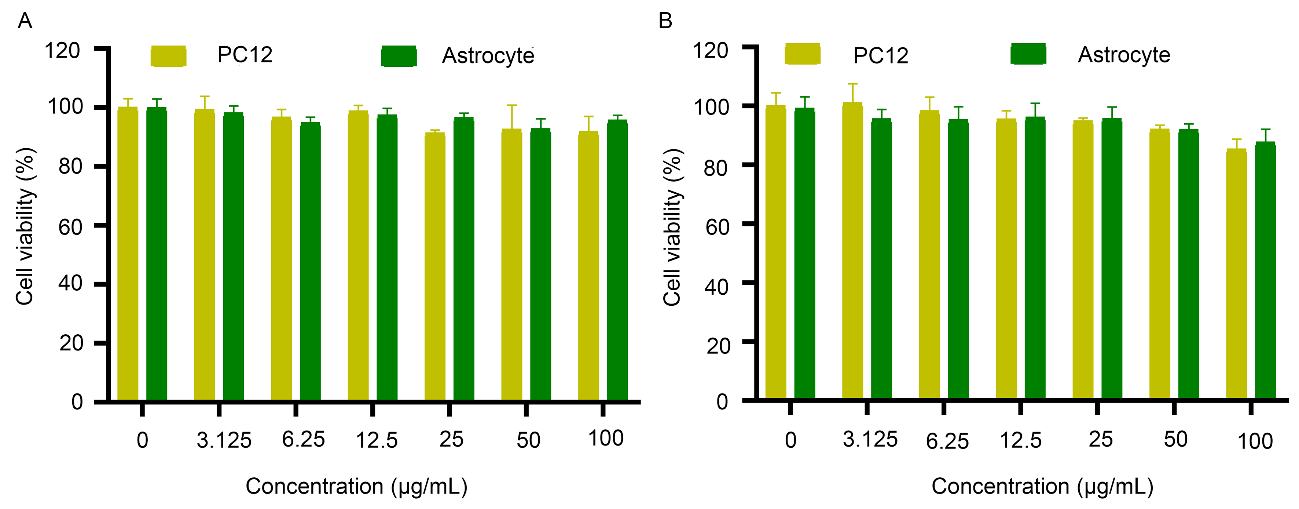


Figure S4. *In vitro* biocompatibility of HA-Se NPs. Viability of PC12 cells and astrocytes incubated with different concentrations of HA-Se NPs for (A) 24 and (B) 48 h. Data are presented as mean ± SD (*n* = 3 for each group).


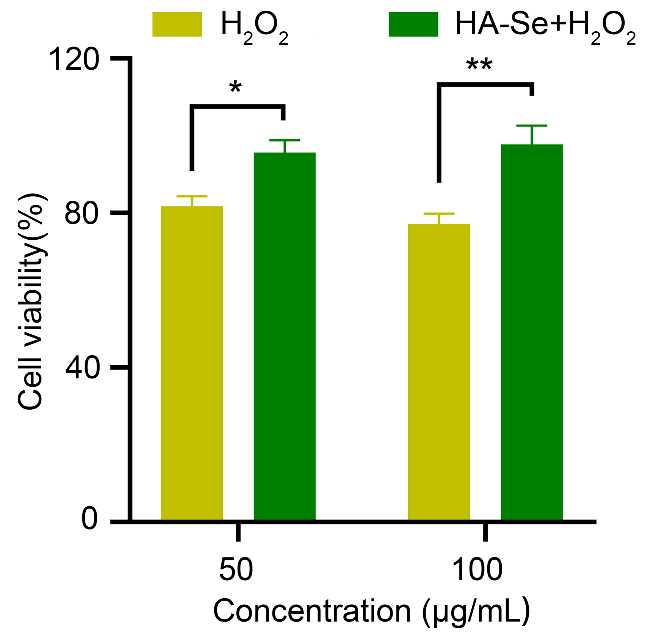


Figure S5. The HA-Se nanoparticles scavenge reactive oxygen species (ROS) to protect astrocytes from oxidative damage. [H_2_O_2_] =100 μM.


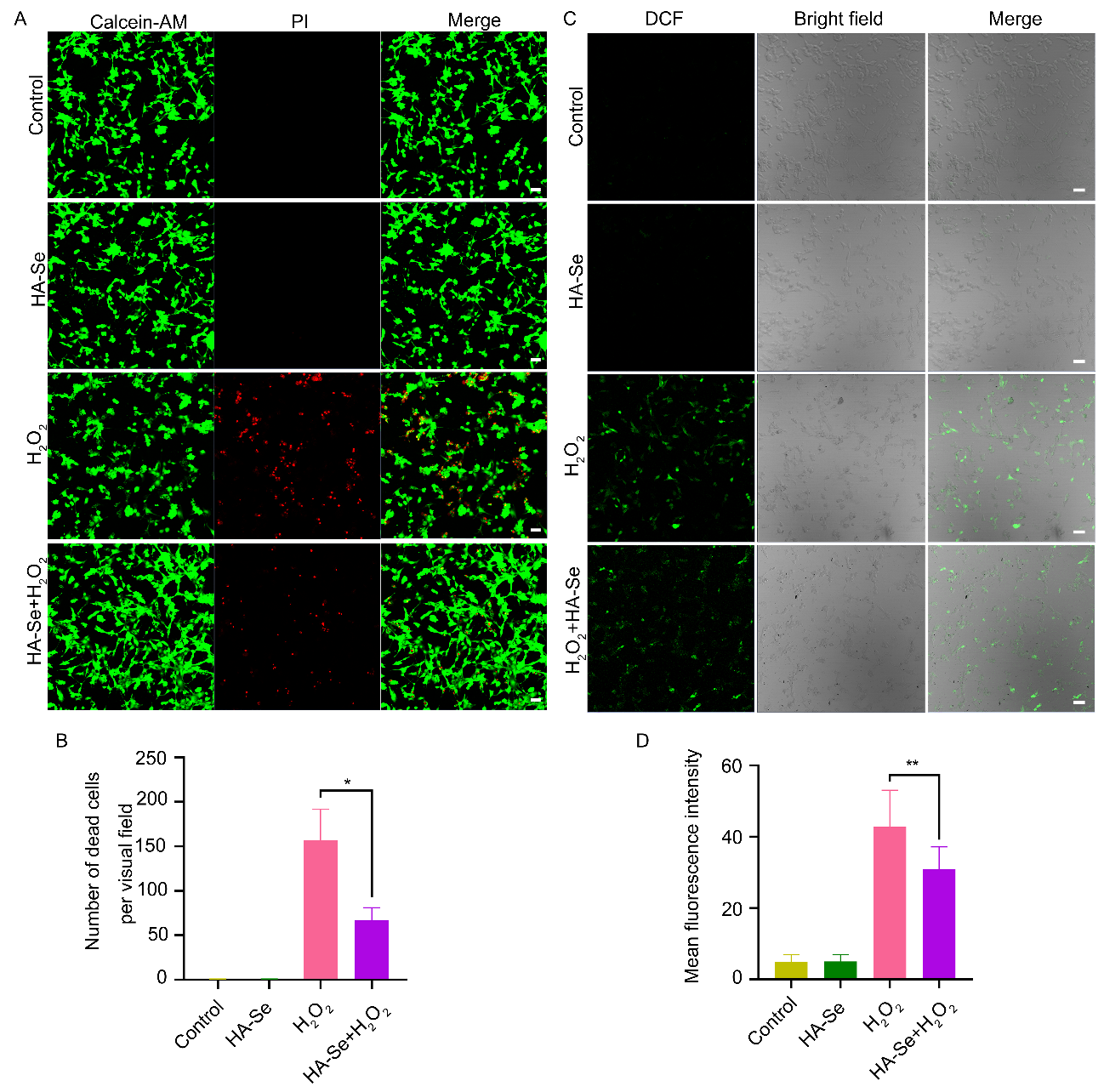


Figure S6. HA-Se NPs scavenge ROS to protect PC12 cells *in vitro*. (A) Live/dead staining of PC12 cells. Scale bar = 20 μm. (B) Quantitative analysis of dead cells. (C) Intracellular ROS levels in PC12 cells were measured using DCFH-DA staining. Scale bar = 20 μm. (D) Quantitative analysis of DCF fluorescence intensity in the cells. ***P*<0.01.

**
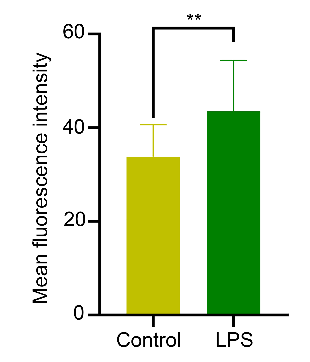
**

Figure S7. Quantitative analysis of the mean fluorescence intensity of CD44 staining in Fig. 4A. ***P*<0.01 compared to the control group.


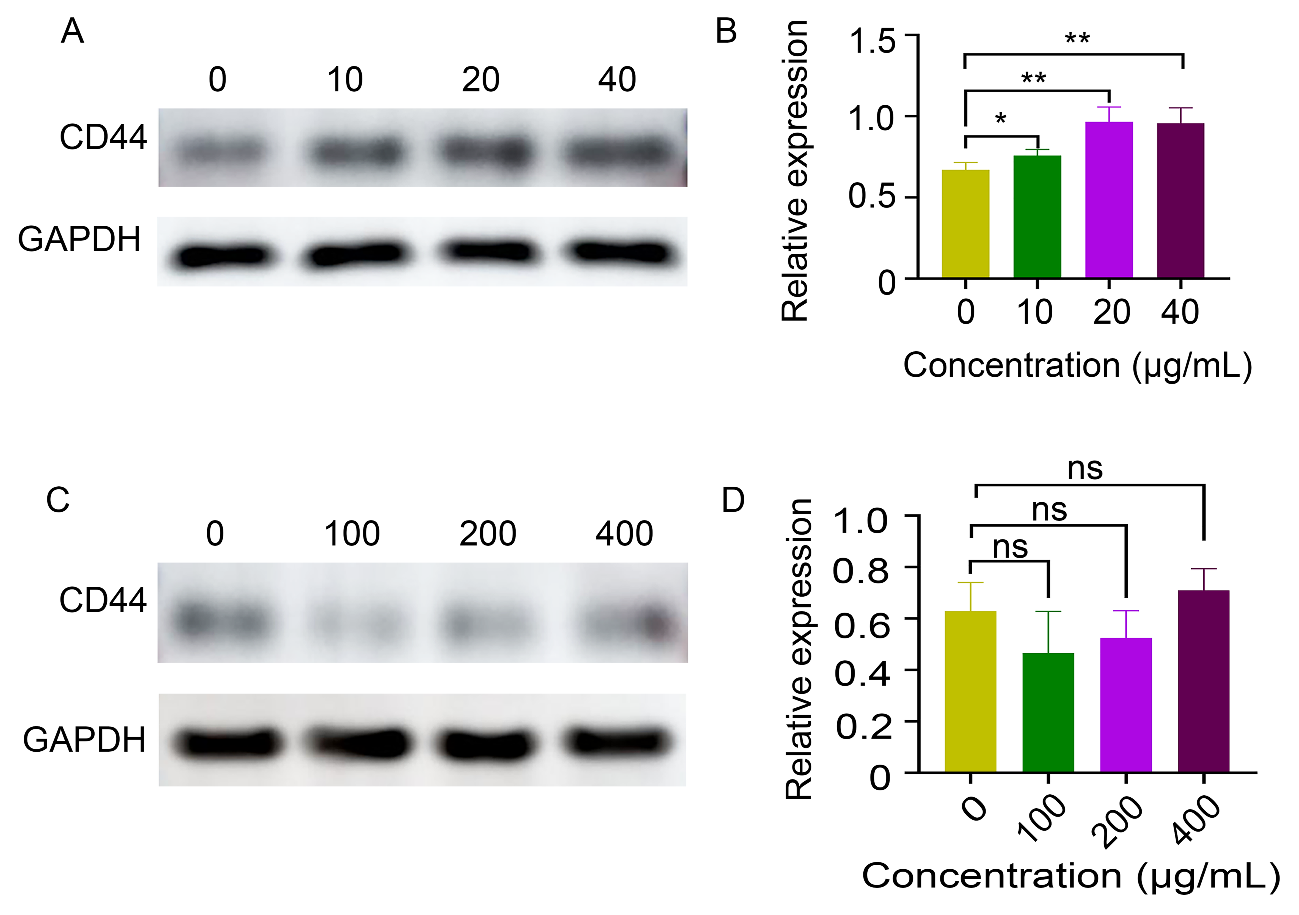


Figure S8. Astrocytes overexpress CD44 upon LPS exposure. Western blot analysis of CD44 in astrocytes upon (A) LPS and (C) glutamate activation. (B, D) Densitometric analysis of CD44 levels based on the data in (A) and (C), respectively. **P*<0.05, ***P*<0.01.

**
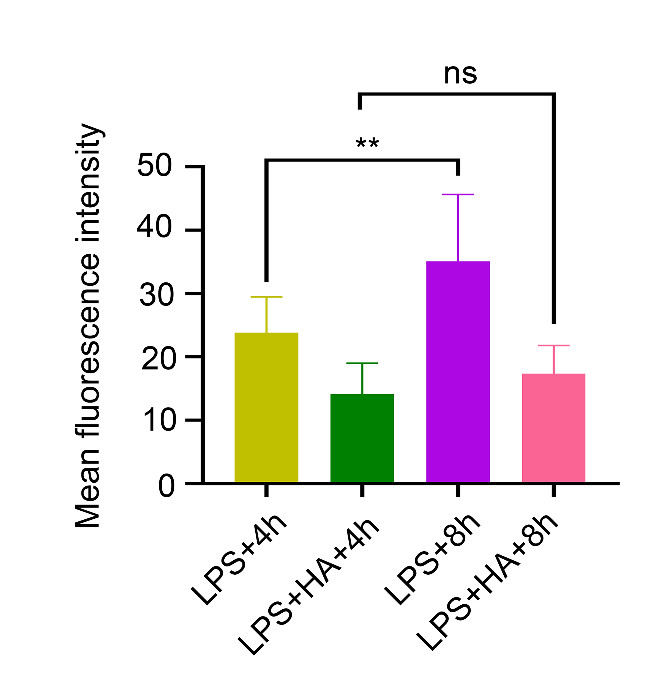
**

Figure S9. Quantitative analysis of the mean fluorescence intensity of Cy5-HA-Se NPs in Fig. 4B. ***P*<0.01 in comparison to the LPS/8 h group.


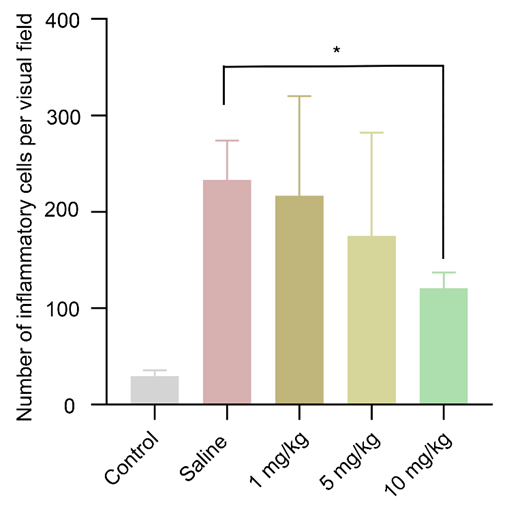


Figure S10. Quantitative analysis of inflammatory cells in Figure 5C, * P < 0.05, in the saline group compared with the 10 mg/kg group.


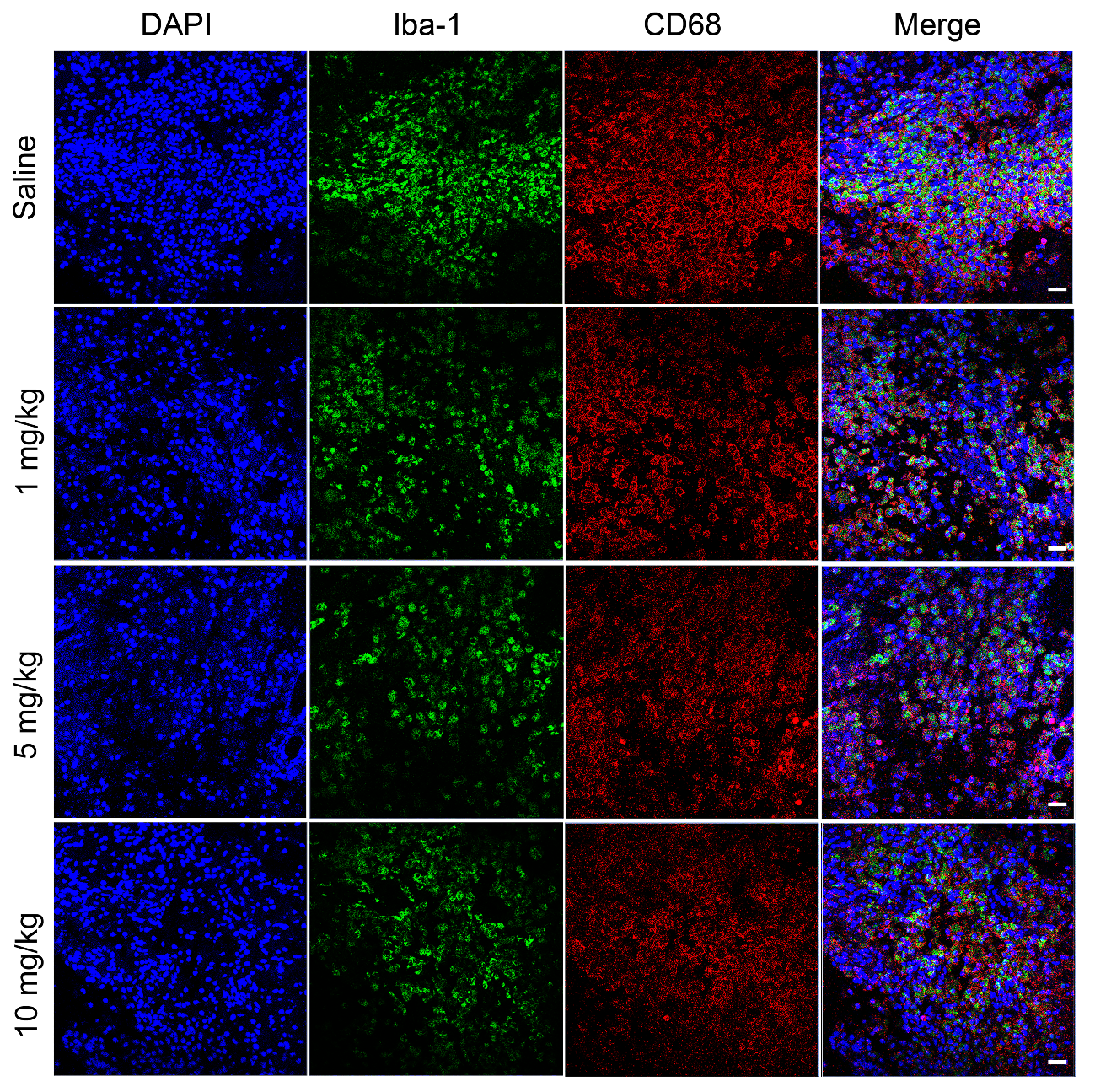


Figure S11. Immunohistochemistry staining of scar tissue labeled with Iba-1 (green) and CD68 (red) 12 weeks after SCI. Scale bar = 50 μm.


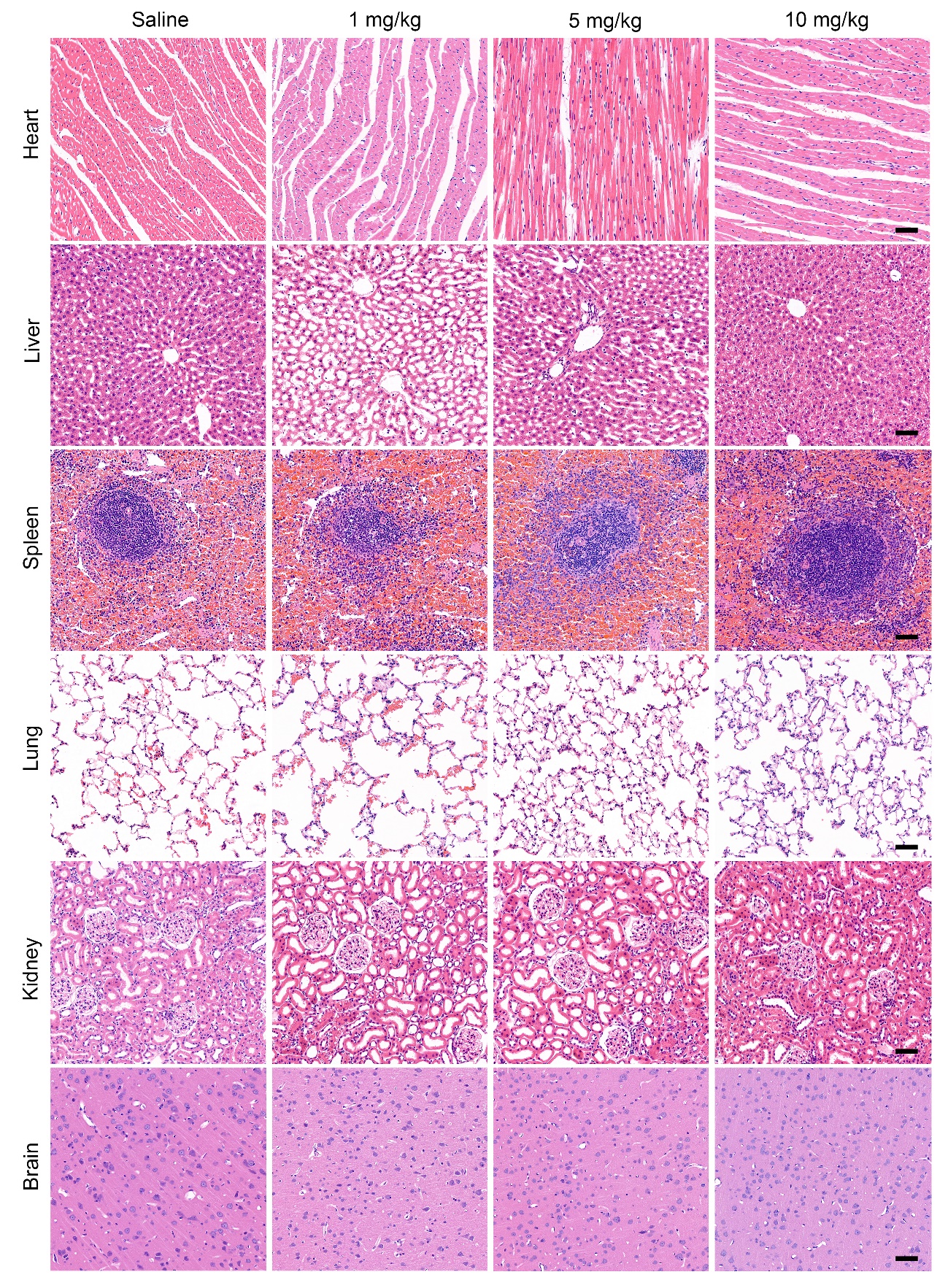


Figure S12. Hematoxylin & eosin (H&E) staining of the major organs in the experimental group.

Table S1. Information on the antibodies used for immunofluorescence (IF) staining

| Antibody | Application | Dilution |
| --- | --- | --- |
| Anti-NF200 | IF | 1:200 |
| Anti-NeuN | IF | 1:500 |
| Anti-Iba-1 | IF | 1:200 |
| Anti-CD68  Anti-GFAP | IF  IF | 1:200  1:1000 |
| Anti-Mouse IgG (H+L) labeled with Alexa-488 | IF | 1:1000 |
| Anti-Rabbit IgG (H+L) labeled with Alexa-546 | IF | 1:500 |
| Anti-Rabbit IgG (H+L) labeled with Alexa-488 | IF | 1:1000 |
| Anti-Mouse IgG (H+L) labeled with Alexa-546 | IF | 1:500 |

Table S2. Antibodies used for western blotting (WB)

| Antibody | Application | Dilution |
| --- | --- | --- |
| Anti-CD44 | WB | 1:1000 |
| Anti-cleaved caspase-3 | WB | 1:1000 |
| Anti-β-Actin | WB | 1:1000 |
| Anti-GAPDH | WB | 1:5000 |
